# Understanding empyema in Qatar: Microbiological profiles, clinical outcomes, and targeted treatment through a retrospective analysis

**DOI:** 10.5339/qmj.2025.110

**Published:** 2025-12-10

**Authors:** Theeb Osama Sulaiman, Mousa Hussein, Mohammed Yaseen, Mansoor Hameed, Merlin Thomas, Abdelnasser Elzouki

**Affiliations:** 1 Department of Chest, Hamad General Hospital, Doha, Qatar; 2 Internal Medicine Department, Hamad General Hospital, Doha, Qatar; 3 Department of Clinical Medicine, Weill Cornell Medicine, Doha, Qatar *Email: theebpalestine.21@gmail.com

**Keywords:** Empyema, Qatar, epidemiology, outcomes, microbiology, mortality

## Abstract

**Introduction:**

Empyema, characterized by the accumulation of pus in the pleural space, poses a significant medical challenge with diverse complications. This study aims to provide a comprehensive overview of the epidemiology, microbiological spectrum, management strategies, and outcomes of empyema patients in Qatar.

**Methods:**

We conducted a comprehensive review of electronic medical records from all hospitals affiliated with Hamad Medical Corporation, the national healthcare system of Qatar, for patients aged 18 years and older diagnosed with empyema between January 1, 2015, and December 31, 2019.

**Results:**

Seventy-five empyema cases were reviewed, predominantly male (89.3%) with a mean age of 44 years. Common comorbidities included diabetes mellitus (30.7%) and a history of malignancy (20%). Pleural fluid was universally exudative, with turbidity in (55.4%) and blood staining in (16.9%). *Streptococcus* (38.7%) and Gram-negative bacteria (29.3%) were the predominant pathogens. All patients received antibiotics, with chest tube insertion as the primary intervention in 74.6%. Video-Assisted Thoracoscopic Surgery (VATS) was performed in 24% and medical thoracoscopy in 1.3%. The mortality rate was significant at 18.6%. Univariate and multivariate analyses indicated that a prior history of pneumonia (*P* = 0.004), pleural effusion/empyema (*P* = 0.024), and malignancy (*P* < 0.036) were significant predictors of mortality.

**Conclusion:**

Streptococci were identified as the predominant pathogen in empyema cases in Qatar, with Diabetes Mellitus being the most common comorbidity. Early antibiotic therapy and chest tube insertion were key to effective management, often reducing the need for surgical interventions. Despite this, mortality remains a significant concern.

## 1. INTRODUCTION

Empyema, the accumulation of pus in the pleural space, arises from infections caused by pyogenic bacteria, fungi, or mycobacteria, typically triggered by adjacent pneumonia, direct inoculation (e.g., from blunt trauma), or hematogenous spread.^1^ The global incidence of empyema is increasing, with a retrospective study reporting a mortality of approximately 15% among hospitalized patients.^2–4^ The microbiological profile of empyema varies by acquisition setting (community, hospital), geographic region, and the patient’s comorbidities, with *Streptococcus* and *Staphylococcus* species commonly implicated.^4,5^ In Qatar, Khan et al. reported bacterial isolates in 47% of empyema fluid cultures, predominantly gram-positive organisms.^6^ Treatment outcomes depend significantly on the chosen management strategy, with surgical debridement being particularly important in advanced cases.^7^

Despite extensive global research, studies on empyema in Qatar are limited, despite its unique demographic, environmental, and healthcare context. To address this gap, we conducted a retrospective analysis of empyema cases from Hamad Medical Corporation (HMC), Qatar’s national healthcare system. Our study characterized microbiological profile, antimicrobial susceptibility patterns, and clinical outcomes in Qatar. By focusing on local clinical, radiological, and microbiological features, this study aims to inform targeted diagnostic and treatment strategies for empyema in Qatar with potential insights for the Arabian Gulf region while acknowledging the limitations of generalizability due to the study sample’s specific context.

## 2. METHODS

HMC operates as the national healthcare system in Qatar, providing medical and surgical services at both secondary and tertiary care levels. This study is a retrospective study focusing on patients admitted to HMC with a confirmed diagnosis of empyema thoracis from January 1, 2015, to December 31, 2019, who meet the study definitions, inclusion, and exclusion criteria listed below. Data collection involved a thorough review of electronic medical records to populate the data collection sheet. Comprehensive examination of history, clinical information, and biochemical data, inclusive of both blood and pleural fluid analysis, along with radiological data, was undertaken and documented. To assess the severity of pneumonia and the risk of complications in these patients, the Pneumonia Severity Index (PSI) score was utilized. The PSI is a well-established clinical scoring system that has been extensively validated and demonstrates high accuracy in predicting mortality, intensive care requirement, and adverse outcomes, including empyema.^8^ The lead research investigators diligently maintained the quality of data, ensuring completeness, verification, validation, accuracy, security, and confidentiality throughout the study. This study was approved by the HMC Institutional Review Board (approval no.: MRC-01-20-524), and the requirement for informed consent was waived due to the retrospective nature of the study.

### 2.1 Study definitions

Empyema thoracis: Patients were classified as “definite empyema” based on one or more of the following criteria: the presence of microorganisms in pleural fluid, Gram stain or culture, pleural pH less than 7.2 associated with radiographic features of empyema, or frank pus in the pleural space at the time of either diagnostic or therapeutic pleural interventions.^4^

### 2.2 Inclusion criteria

All patients above the age of 18 years are diagnosed with empyema.

### 2.3 Exclusion criteria

Tuberculous and malignant empyema were excluded from this study to ensure a more homogeneous patient population and to allow for focused analysis of the microbiological profiles and treatment outcomes of pyogenic empyema.

### 2.4 Statistical analysis

Categorical and continuous values were expressed as frequency (percentage) and mean ± SD or median and interquartile range (IQR) as appropriate. Descriptive statistics were used to summarize demographic, laboratory, and clinical characteristics of the patients. The primary outcome was to evaluate and assess the clinical characteristics, microbiology, and management options with their outcome and complication rates. Associations between two or more qualitative variables were assessed using the chi-square test or Fisher’s exact test as appropriate. Quantitative variables and the mean between the two independent groups were analyzed using the unpaired t-test and the Mann-Whitney *U* test. The relationship between two quantitative variables was examined using Pearson’s correlation coefficient. Multivariate analysis was done using a stepwise binary logistic regression model. The results were presented with the associated 95% confidence interval. All *P* values presented are two-tailed, and *P* values <0.05 are considered statistically significant. All Statistical analyses were performed using the statistical package SPSS 19.0 (SPSS Inc., Chicago, IL).

## 3. RESULTS

We performed a comprehensive analysis of 75 patients diagnosed with empyema during the study’s inclusion period.

### 3.1 Demographics and clinical presentation

The mean age of the patients was 44 years, with a predominance of males, who represented 89.3% of the cohort compared to 10.6% females. Non-smokers accounted for 49.2% of the patient population, while 84.1% reported no alcohol consumption. Among the observed comorbidities, Diabetes Mellitus was the most common, affecting 23 patients (30.7%), followed by hypertension in 19 patients (25.3%) and coronary artery disease in 7 patients (9.3%). A history of malignancy was noted in 20% of the patients, with each of the following types occurring at 13%: lung cancer, esophageal cancer, breast cancer, and osteosarcoma. Table 1 presents demographic data and clinical comorbidities recorded in our patient cohort.

The clinical symptoms observed in our study patients at hospital presentation are summarized in Table 2. The median PSI score was 78 (56–118), and the median duration of symptoms before presentation was 3 (1–6) days. Imaging results showed that all patients had an effusion on their Chest X-ray, with 40% (*n* = 30) undergoing Chest CT scans. Septated pleural effusion was found in 32% (*n* = 24) of the patients, and pneumothorax was noted in 9.3% (*n* = 7) of cases.

### 3.2 Management of patients

Patient management is centered on a combination of antibiotics and thoracic interventions. Antibiotic administration, guided by pleural fluid culture and sensitivity, was universal among our patients. The most frequently prescribed antibiotics included Ceftriaxone (*n* = 20; 26.7%), Amoxicillin/Clavulanic acid (*n* = 18; 24%), Piperacillin-tazobactam (*n* = 12; 16%), and Meropenem (*n* = 9; 12%). Less commonly used agents comprised Vancomycin in eight patients, Clindamycin in six patients, and Ertapenem in five patients. Antifungal agents, namely Caspofungin and Anidulafungin, were administered to two patients.

Table 3 outlines the modalities of intervention employed in our patient cohort. Notably, chest tube insertion served as the initial intervention in 74.6% of cases, with 30 patients (40%) receiving wide-bore chest tubes (external diameter > 14 Fr) and 26 patients (34.6%) receiving small-bore chest tubes (external diameter < 12 Fr). Among patients who managed with a chest tube, only 7.14% required subsequent Video-Assisted Thoracoscopic Surgery (VATS). VATS itself was the primary intervention in 24% of patients, while medial thoracoscopy was conducted in only one patient (1.3%). The average time from hospital admission to thoracic intervention was 4.3 ± 5 days.

### 3.3 Results of blood and pleural fluid investigations

Regarding pleural fluid characteristics, the majority presented with a turbid appearance (55.4%), followed by blood-stained effusions (16.9%). Light’s criteria were applied to distinguish between transudative and exudative pleural effusions. All patients fulfilled the criteria for exudative pleural fluid. Microbiological analysis reported *Streptococcus* species to be the most frequently isolated pathogens from the pleural fluid, occurring in 38.7% of patients. Table 4 summarizes the blood and pleural fluid analyses of the study participants.

Regarding the outcome of our study cohort, the majority of patients, comprising 34 individuals (45.3%), experienced complete resolution during the follow-up period. Conversely, 26 patients (34.6%) did not undergo follow-up imaging to document resolution. Only one patient (1.3%) experienced complication events, leading to the development of a Bronchopleural fistula, whereas mortality was documented in 14 patients (18.6%). Among the 34 patients (45.3%) who achieved complete resolution, the most frequently isolated pathogens were *Streptococcus* in 15 patients (44.1%), followed by *Staphylococcus* in six patients (17.6%), and *Klebsiella* species in five patients (14.7%). Chest tube insertion was the exclusive intervention in 21 patients (61.7%), while 12 patients (35.2%) required VATS, and one patient (2.9%) underwent medical thoracoscopy. In the group of patients who succumbed to the condition, *Staphylococcus* species were the predominant isolated organisms in six patients (42.8%), followed by *Streptococcus* in five patients (35.7%) and *Escherichia coli* in two patients (14.2%). Regarding interventions, small-bore chest tubes were employed in seven patients (50%), while six patients (42.8%) required large-bore chest tubes, and one patient (7.1%) underwent VATS.

To determine which factors were associated with death, we performed a multivariable analysis to assess the impact of patient and procedure characteristics on mortality. On univariate analysis, the odds of mortality were increased with age (54.1 ± 14.3 vs. 41.6 ± 14.9; *P* = 0.01), higher PSI score (184.5 vs. 72; *P* = 0.012), prior history of pneumonia or history of pleural effusion/empyema (*P* = 0.001; *P* < 0.001 respectively), history of malignancy (*P* = 0.002), and bloody pleural fluid appearance (*P* = 0.021). On multivariable analysis, the prior history of malignancy, history of pneumonia, or history of pleural effusion/empyema were associated with higher odds of death (Table 5).

## 4. DISCUSSION

The incidence of adult empyema has been reported to range between 6 and 12 cases per 100,000 population in international studies, reflecting a rising global trend.^9^ Data from the Middle East remains limited, with a Saudi hospital-based study reporting approximately 23 cases per 100,000 admissions.^10^

Our study stands as the initial exploration into the demographic data, microbiology, and management of empyema in Qatar, and it stands as the most recent research in the Gulf Cooperation region. The predominant gender in our cohort was male, constituting 89.3%, and the average age of patients was 44 years. This age profile contrasts with other empyema studies reporting mean ages of 59.4 to 64 years, underlining the unique demographic characteristics of the country.^11–13^ The retrospective analysis highlighted diabetes mellitus as the most prevalent associated condition in our patients, accounting for 30.7%. This prevalence surpasses figures reported in analogous studies, which typically range from 8.1% to 11%.^14,15^ This variance might be attributed to most of our patients belonging to the Indian subcontinent, where the prevalence of diabetes mellitus is notably higher.^16^

In terms of microbiology, all patients within our cohort exhibited a positive pleural fluid culture. The most frequently isolated microorganism, in descending order of prevalence, was *Streptococcus* species (38.7%), succeeded by gram-negative bacteria (29.3%), and *Staphylococcus* species (28%). Notably, other studies also consistently report the predominance of Streptococci bacteria.^2,17^ However, in contrast to our cohort, Staphylococci species have emerged as the subsequent isolated organism after *Streptococcus*. This distinction could be attributed, in part, to the low occurrence of prior thoracic procedures and the relatively lower rate of drug abuse in Qatar.^18^ All patients in our cohort received antibiotics based on pleural fluid culture and sensitivity. Regarding interventions, initial management for 56 patients involved chest tube drainage. Remarkably, 52 of these patients (92.8%) did not require further intervention, demonstrating the effectiveness of chest tube drainage as the sole modality. This contrasts with other studies reporting a lower treatment effectiveness rate with chest tube alone (40%; 48.5%).^19,20^ The likely explanation lies in the early administration of antibiotics in our patients, coupled with regular chest drain flushing to prevent blockages. Notably, none of our patients underwent intrapleural fibrinolysis.

VATS was employed in 22 patients (29.3%), and surprisingly, none of these patients required open thoracotomy for decortication. In contrast, Chambers et al. reported conversion rates to open decortication ranging from 0% to 3.5% in early-stage empyema and 7.1% to 46% in the late stage.^21^ Another study documented a high conversion rate from VATS to open procedures, reaching 57.89%.^22^ The elevated effectiveness of VATS in our study is likely attributable to younger patients with fewer adhesions and early-stage empyema, characterized by exudative and early fibrinopurulent stages.

Our cohort had a mortality rate of 18.6%, falling within the lower spectrum of mortality rates reported in other studies, ranging from 18% to 60%.^23–25^ Among patients who succumbed, *Staphylococcus* species were the most frequently isolated organisms (42.8%), followed by *Streptococcus* (35.7%) and *Escherichia coli* (14.2%). In our study, the primary determinants of mortality were significantly elevated in those with advanced age, higher PSI score, a history of pneumonia, pleural effusion/empyema, malignancy, and bloody pleural fluid. Multivariable analysis demonstrated that a history of malignancy, pneumonia, or pleural effusion/empyema was significantly associated with decreased survival. Findings of advanced age and prior history of malignancy were consistent with findings from other studies.^26–28^

The RAPID score, comprising older age, elevated blood urea nitrogen levels, low serum albumin levels, hospital-acquired infection, and the absence of purulent fluid, is extensively utilized in the assessment of pleural infections and has been correlated with poor prognosis at 3 months.^29^ Among these components, only advanced age was found to be significantly associated with adverse outcomes in our study, as the other factors did not show statistically significant differences between the survivor and non-survivor groups. Other factors identified in the literature as correlating with poor survival include alcoholism,^26^ end-stage renal disease,^30^ hypoalbuminemia,^31^ and polymicrobial etiology.^32^

However, our study has several limitations. First, the retrospective nature of the design may introduce bias, and the fact that the research was conducted in a single country might restrict the generalizability of the results. Second, although we excluded malignant pleural effusion, around 20% of the study participants had a prior history of malignancy. These cases were thoroughly reviewed to confirm that the empyema was of pyogenic origin and unrelated to active cancer. Nevertheless, a history of malignancy could still act as a confounding factor, since previous cancer treatments or associated comorbidities might affect infection susceptibility or clinical outcomes. This should be taken into account when interpreting the findings, and future research could benefit from stratifying patients according to their cancer history. Lastly, although previous episodes of empyema were found to be a significant risk factor for mortality in our multivariable analysis, detailed information about the underlying cause of those previous episodes was often incompletely documented in the retrospective records. As a result, we could not reliably determine whether the prior and current episodes shared the same etiology. Including such data without adequate verification could introduce misclassification bias and compromise the validity of our findings. Despite these constraints, the study imparts valuable insights into the epidemiology and management of empyema in Qatar. The authors advocate further research to enhance our understanding of empyema risk factors and mortality and to devise more effective treatment strategies.

## 5. CONCLUSION

Streptococci are the predominant bacterial cause of empyema in Qatar and are frequently associated with diabetes mellitus as a significant comorbidity. The mortality rate observed in our cohort aligns with global trends. Key factors linked to increased mortality include a prior history of malignancy, pneumonia, and previous pleural effusion or empyema. Given the limitations inherent in retrospective designs, future prospective multicenter studies are warranted to validate these findings and explore additional prognostic indicators.

## ETHICAL APPROVAL

The study was approved by the Institutional Review Board, Hamad Medical Corporation, under MRC research protocol no: 29 0120524.

## FUNDING

The research was funded by the Medical Research Center (MRC), Hamad Medical Corporation, Doha, Qatar

## CONFLICT OF INTEREST

This manuscript is an original one. It has not been published or considered for publication elsewhere. None of the authors has any financial or conflict of interest in publishing this manuscript. The manuscript has been seen and agreed upon by all authors.

## Figures and Tables

**Table 1.** Baseline characteristics of patients.

Clinical characteristic	Number of patients, *N* (%)
Mean age, years	44 ± 15.6
**Gender**	
Males	67 (89.3%)
**Smoking**	
Non-smoker	31 (49.2%)
Ex-smoker	14 (22.2%)
**Alcohol**	
Alcohol consumer	6 (9.5%)
Ex-alcohol consumer	4(6.3%)
**Comorbidities**	
Diabetes mellitus	23 (30.7%)
Hypertension	19 (25.3%)
Neoplastic disease	15 (20%)
Prior history of thoracic procedure	14 (18.7%)
Prior history of pneumonia	13 (17.3%)
History of other known medical conditions*	23 (30.7%)
Prior history of pleural effusion/empyema	7 (9.3%)
Prior history of any relevant surgery or illness	5 (6.7%)
Chronic kidney disease	3 (4%)
History of cerebrovascular disease	3 (4%)
Chronic liver disease	2 (2.7%)
History of asthma	1 (1.3%)
History of COPD	1 (1.3%)
Chronic heart failure	1 (1.3%)

**Table 2.** Presenting symptoms of our patients.

Presenting symptom	Number of patients, *N* (%)
Shortness of breath	44 (60.3%)
Chest pain	41 (54.7%)
Fever	39 (52.7%)
Cough	36 (48%)
Weight loss	13 (17.3%)
Altered mental status	10 (13.3%)
Loss of appetite	9 (12%)
Night sweats	5 (6.7%)
Hemoptysis	3 (4%)

**Table 3.** Interventional procedures for management of empyema.

Intervention	Number of patients, *N* (%)
Small-bore chest tube	26 (34.7%)
Wide-bore chest tube	30 (40%)
VATS	22 (29.3%)
Medical thoracoscopy	1 (1.4%)

**Table 4.** Serum and pleural fluid characteristics of our patients [The data are represented with mean ± SD and range (IQR)].

Variable	
Serum parameters	N, mean ± SD, and range (IQR)
White Blood Cell (WBC) Count	74, 15.2 ± 7.3
Lymphocyte %	75, 7.3 (4.9–11.4)
Neutrophil %	74, 78.3 ± 14.6
C-reactive protein	71, 256 (130–328)
Procalcitonin	57, 0.9 (0.36–2.7)
Lactate dehydrogenase (LDH)	32, 218 (176–315)
Serum protein	74, 62.8 ± 9.4
Blood urea nitrogen (BUN)	75, 5.9 (4.1–13.2)
Albumin	75, 23 (19–29)
PSI score	43, 78 (56–118)

**Pleural fluid analysis**	***N* (%)**

**Appearance**	
Turbid	36 (55.4%)
Bloody	11 (16.9%)
Milky	6 (9.2%)
Straw	7 (10.9%)
Brown	4 (6.2%)
**Microbiology**	
*Streptococcus*	29 (38.7%)
*Staphylococcus*	21 (28%)
Enterococcus	6 (8%)
Gram-negative bacteria	22 (29.3%)
*Pseudomonas*	6 (27.2%)
*Klebsiella pneumoniae*	5 (22.7%)
*Enterobacter cloacae*	3 (13.6%)
*Escherichia coli*	3 (13.6%)
*Acinetobacter baumannii*	3 (13.6%)
*Candida albicans*	2 (2.6%)

**Table 5.** Association between various potential predictors with mortality (multivariate logistic regression analysis).

Predictor variables	Adjusted odds ratio (OR) (95% CI)	*P* value
Prior history of pneumonia	12.96 (2.30, 72.93)	0.004
Prior history of Pleural effusion/empyema	11.55 (1.38, 96.46)	0.024
Prior history of neoplastic disease	6.40 (1.13, 36.17)	0.036
